# Pennsylvania State Veterinary Medical Association

**Published:** 1891-09

**Authors:** Robert Gladfelter

**Affiliations:** Cor. Secretary


### EDITORIAL DEPARTMENT.

### PENNSYLVANIA STATE VETERINARY MEDICAL ASSOCIATION.

The semi-annual meeting of the Pennsylvania State Veterinary Medical Association, will be held at the County Medical Association rooms, Coal Exchange Building, Cor., River and Market Sts., Wilkesbarre, Pa., Tuesday, Sept. 8, 1891, at 10 o'clock P. M.

The general order of business will be conducted, and reports of the following committees:

Committee on Legislation, Dr. W. A. Hoskins, chairman; Committee on Intelligence and Education, Dr. R S. Huidekoper, chairman; Committee on Sanitary science and Police, Prof. W. L. Zuill, chairman.

Essays by Dr. W. H. Hoskins, "The Veterinarian as a Sanitarian"; Dr. S. E. Weber, "Pleurisy and its Complications"; Dr. J. F. Butterfield, "Results of Laryngitis."

ROBERT GLADFELTER,

*Cor. Secretary.*

